# The Influence of the Variety, Vineyard, and Vintage on the Romanian White Wines Quality

**DOI:** 10.1155/2016/4172187

**Published:** 2016-10-19

**Authors:** Anamaria Hosu, Veronica Floare-Avram, Dana Alina Magdas, Ioana Feher, Mihai Inceu, Claudia Cimpoiu

**Affiliations:** ^1^Faculty of Chemistry and Chemical Engineering, Babes-Bolyai University, 11 Arany Janos, 400028 Cluj-Napoca, Romania; ^2^National Institute for Research and Development of Isotopic and Molecular Technologies, 67-103 Donat, 400293 Cluj-Napoca, Romania

## Abstract

The wine is one of the most consumed drinks over the world, being subjected to falsification or adulteration regarding the variety, vintage, and geographical region. In this study, the influence of different characteristics of wines (type, production year, and origin) on the total phenolic content, total flavonoids content, antioxidant activity, total sugars content, pH, and ^18^O/^16^O isotopic ratio was investigated. The differentiation of selected wines on the basis of tested parameters was investigated using chemometric techniques, such as analysis of variance, cluster analysis, and principal component analysis. The experimental results are in agreement with other outcomes and allow concluding that variety and vineyard have the major influence on the studied parameters, but, also, statistical interaction effect between year and vineyard and year and variety is observed in some cases. The obtained results have demonstrated that these parameters together with chemometric techniques show a significant potential to be used for discrimination of white wines.

## 1. Introduction

The wine is one of the most consumed drinks over the world. Consequently, the wine is one of the liquid products that are subjected to falsification or adulteration regarding the variety, vintage, and geographical region. The wine origin has been considered to be a quality indicator and wine consumers often require information on the provenance [1]. In the literature, various classifications of wines based on their variety, vintage, and geographical origin using different criteria, such as the phenolic compounds [2, 3], combination of polyphenols and antioxidant activity [4], isotope ratios [5, 6], volatile aroma compounds [7, 8], and amino acids [9], were reported.

The composition and concentration of phenolics in wine depend on the type of grape used for wine production, the procedures employed for winemaking, and the chemical reactions that occur during the aging of wine [10]. Polyphenols control the color, aroma, bitterness, and taste, acting as photoprotective pigments and antioxidants and playing an essential role in wine quality [11]. Also, phenolic composition of wines influenced their color stability and browning reactions and higher polyphenolic content helps to stabilize the wine against detrimental temperature effects [12]. Phenolic compounds can be successfully used for wine authenticity assessment as they are characteristic for the type of wine and can provide information on geographical origin [13]. Polyphenolic compounds are classified as flavonoids (flavanols, flavonols, dihydroflavonols, and anthocyanins) and nonflavonoids (hydroxybenzoic and hydroxycinnamic acids, stilbenes, and phenolic alcohols), the last class representing the majority of polyphenolic compounds of white wines. Nevertheless, flavonoids have a greater impact on the structure and color of wine compared to nonflavonoids. The flavonoids are found in skins, seeds, and stems of white grapes and represent about 25% of total phenolic content (TPC) in white wines [14]. For European white wines grown under cool climate conditions, the presence of flavonoids is considered undesirable because the typical cultivar aroma is reduced [15].

The polyphenolic compounds show antioxidant effect that is related to the health benefit of moderate wine consumption. The antioxidant activity depends on the phenolic profile of wine because each polyphenol contributes differently to the wine's activity. During storage, oxidation of polyphenolic compounds leads to changes in the levels of antioxidants in wine, as a consequence of changes in the redox equilibrium, being essential because of its influence on the organoleptic characteristics and because of its importance regarding its antioxidant potential [16]. Several* in vitro* and* in vivo* methods have been used to measure the antioxidant activity in wines, such as Oxygen Radical Absorbance Capacity (ORAC) method, 2,2-azinobis(3-ethylbenzothiazoline-6-sulfonic acid) (ABTS), 1,1-diphenyl-2-picrylhydrazyl (DPPH), Trolox Equivalent Antioxidant Capacity (TEAC), Ferric Reducing Ability of Plasma (FRAP), and N,N-dimethyl-p-phenylenediamine (DMPD) [17, 18].

Besides polyphenolic compounds, sugars are of great importance for the organoleptic quality of wines. The positive effect of total sugars content (TSC) is due to the changing gustatory structure and fullness and body and softening astringency of wines [19]. The influence of sugars on wine characteristics depends on their total quantity, structure, composition, and distribution, appearing as relevant variables differing among segments and showing some differences between varieties [20]. Also, the TSC is influenced by climate and geographical region.

One of the most often used parameters for monitoring the maturation of wines is pH, which is a measure of the likelihood and speed of occurrence of pH-dependent reactions. The major roles of pH regarding the quality of wine are as follows: microbial stability of wines [21] and perception of acidity and its impact on fruit flavor and acid taste and sugar-acid balance of wines [22]. Winemakers can adjust the pH of the wine by organic acids or ion exchange materials or by use of rootstock [22], but at increased cost of input [23].

Another marker for vintage and geographical origin is the stable oxygen isotope ratios (^18^O/^16^O) of wine water [24]. The main factor that affects the oxygen isotopic ratio from plant water is the available water source which, in most cases, is the groundwater that basically results from precipitations. Additionally, the climatic conditions, which already influenced the isotopic composition of precipitations, and, also, the evaporation and transpiration processes always produce enrichment in heavy isotopes of plant water in comparison with groundwater [25]. Beside these, other factors that affect the oxygen isotopic ratios of wine water are as follows: meteorological conditions, soil type, and date of harvesting.

The aim of this study was to evaluate the influence of different characteristics of wines (variety, vintage, and vineyard) on their properties. Chemometric techniques such as analysis of variance (ANOVA), cluster analysis (CLU), and principal component analysis (PCA), which provide the possibility of systematizing the obtained data from different analytical techniques [26, 27], were used in order to investigate the differentiation of selected wines with respect to the type, the geographical origin, and the production year. On the basis of our knowledge, this is the first study of the influence of these characteristics on the quality of Romanian white wines.

## 2. Experimental

### 2.1. Materials and Methods

Twenty-seven commercial wine samples from different vineyards of Romania, produced in 2008, 2009, and 2010 vintages, were investigated in this study. The chosen wine cultivars for this work were Fetească Albă, Sauvignon Blanc, and Riesling. All the wines were purchased from local wine shop.

#### 2.1.1. Spectrophotometric Measurements

All spectrophotometric measurements were made using Spectrophotometer T80+ (PG Instruments). All measurements and analyses were carried out in triplicate and the data were presented as the means ± standard deviations.

*(1) Total Phenolic Content (TPC).* Folin-Ciocalteu method was applied for determination of TPC [4]. First, 0.3 mL of sample was mixed with 1.5 mL of Folin-Ciocalteu reagent and, after 5 minutes, 1.2 mL of sodium carbonate 0.7 M was added. Sample was incubated at room temperature, in dark place for 2 h, and the absorbance was measured at 760 nm. The results were expressed in *μ*g of gallic acid/mL of wine on the basis of calibration curve obtained by the same procedure using standard solution of gallic acid (10–250 *μ*g/mL).

*(2) Total Flavonoids Content (TFC).* First, 1.2 mL of wine was mixed with 0.6 mL of NaNO_2_ (5%) and, after 5 min, 1.2 mL of AlCl_3_ (10%) was added. Then, after 5 min, 2 mL of NaOH 0.1 M was added. The absorbance was measured at 430 nm after 10 min [4]. Standard solution of rutin (10–125 *μ*g/mL) was used for the calibration curve and the results were expressed in *μ*g of rutin/mL of wine.

*(3) Antioxidant Activity (AOA).* The determination of antioxidant activity was done by DPPH assay [4]. Aliquots of 0.25 mL of wine diluted three times with distilled water were added to 3.0 mL of 0.09 mg/mL methanolic solution of DPPH. The absorbance of the reaction mixture was measured at 517 nm, after 20 min. Calibration was performed using vitamin C as standard, in the concentration range of 0.150–0.275 mg/mL, following the same procedure. The obtained calibration curve (*y* = 1.471*x*, *r* ^2^ = 0.9912) was used for antioxidant activity calculation.

*(4) Total Sugars Content (TSC).* The TSC in wines was determined by Dubois method [28]. The dry and semidry wines were diluted 50 times and the semisweet wines were diluted 300 times. 0.1 mL of diluted wine was mixed with 0.5 mL of 5% aqueous solution of phenol, and then 2.5 mL of concentrated sulfuric acid was added. The absorbance was measured at 490 nm after 20 minutes. The total sugar content was calculated on the basis of calibration curve obtained by the same method using D-glucose as standard.

#### 2.1.2. pH

The pH of each wine was measured using a pH-meter InoLab pH 7110.

#### 2.1.3. Isotopic Measurements

The measurements were made using carbon dioxide [29]. For *δ* ^18^O determination, the equilibration of CO_2_ with the wine water was carried out by introducing 5 mL of the wine sample into a calibrated sample bottle and cooling it down to −80°C. After venting the bottle, the carbon dioxide was introduced at a pressure value of about 600 Torr, the bottle being placed in the thermostatically controlled water bath at 25°C. The isotopic equilibrium is reached overnight (around 16 h). Before measuring *δ* ^18^O, after the equilibration step, the carbon dioxide contained in the bottles was extracted and cryogenically purified.

The measurements of ^18^O/^16^O isotopic ratio were performed by Delta V Advantage Isotope Ratio Mass Spectrometer (ThermoFinnigan, Bremen, Germany) by dual inlet method. The abundance of stable isotopes was presented in delta notation.

^18^O*/* ^16^O isotope ratios determinations were performed versus laboratory standard water calibrated using reference material (RM) water, Puerto Rico laboratory reference, W-39500, IAEA, Vienna, with *δ* ^18^O_VSMOW_ = −1.52 ± 0.07‰. *δ* ^18^O value is expressed according to the relation

(1) $$\begin{array}{c} \delta^{18}O=1000\times \frac{\left(R_{sample}-R_{ref}\right)}{R_{ref}}, \end{array}$$

where *R*_sample_ and *R*_ref_ are ^18^O/^16^O isotope ratios of the sample and of the carbon dioxide used as the reference gas. The reproducibility of the measurements was 0.2‰.

### 2.2. Statistics

The experimental results were subjected to ANOVA, cluster analysis, and principal component analysis using STATISTICA 10 software (StatSoft Inc., Tulsa, USA). The ANOVA analysis was performed in order to reveal the differences between geographical region, production years, and wine variety and to determine which variables affect the response of the investigated problem. The differences were considered to be significant at the level of *p* < 0.05 for 95% probability. According to these *p* values, the characteristics of wines were ranked, the best rank being given by the lowest *p* value [30]. The cluster analysis (CLU) was performed for grouping the cases instead of variables. The CLU is used as a feature for clustering variables that identifies the key variables which explain the principal dimensionality in the data [31]. Basic chemometric characterization of the investigated wine samples is made by principal component analysis (PCA), which depicts a natural grouping of the studied objects as well as the variables (descriptors) in multidimensional space without forcing the objects or variables to be organized according to some classification principle [32]. PCA is a powerful technique that reduces the dimension of original data matrix by retaining the maximum amount of variability. Before carrying out the PCA two important tests are made: Kaiser-Meyer-Olkin (KMO) Measure of Sampling Adequacy and Bartlett's Test of Sphericity. The KMO statistic varies between 0 and 1. Bartlett's Test measures the null hypothesis that the original correlation matrix is an identity matrix, being significant for *p* values less than 0.05.

## 3. Results and Discussion

Three white wine sorts were investigated in this study, namely, Fetească Albă (traditional Romanian wine sort), Sauvignon Blanc, and Riesling. The selected wines were produced in four different Romanian regions during three consecutive years. All experimental results are presented in Table 1 as means ± standard deviations (*n* = 3).

### 3.1. Total Polyphenolic Content (TPC)

In the case of Sauvignon Blanc wines, a difference between the two vineyards from Oltenia region is observed. Thus, TPC for 2009 is 174.8 *μ*g/mL in the case of vineyard I and 257.4 *μ*g/mL of wine from vineyard II. This variation can be attributed to the different winemaking conditions used in these wineries. The polyphenols are present in the solid part of grapes and their extraction is determined by winemaking conditions, such as grapes squeezing procedure, pH, and temperature. These results are in agreement with Gómez-Míguez et al. (2007) confirming that winemaking techniques have a high influence on phenolic content [33].

Regarding Fetească Albă wines, the highest amount of TPC is found in wines from region of Moldavia (413.3 *μ*g/mL in 2009), while, for Fetească Albă wines from Muntenia region, in the same year, TPC was 230.7 *μ*g/mL and 260.0 *μ*g/mL, respectively. In the case of wines made by wineries from Muntenia region, no notable differences in the TPC are observed, the TPC values being comparable for the same production year and the highest TPC value being for 2010.

Concerning Riesling wines, a descending trend from 2008 to 2010 is observed, the TPC values decreasing from 445.2 *μ*g/mL for 2008 to 276.3 *μ*g/mL for 2010. The biosynthesis of polyphenols is mostly influenced by sun exposure and temperature, so the wines produced from the grapes cultivated in warm and sunny areas will have the highest quantity of polyphenolics [34]. Thereby, the low TPC value for 2010 could be due to the fact that this year was a rainy year in Transilvania region.

There are differences between the regions, Oltenia, Muntenia, Transylvania, and Moldavia. Thereby, in 2008 the highest TPC value is found in wines from the region of Transylvania (445.2 *μ*g/mL) that is almost twice as high as those from Oltenia and Muntenia regions. In 2009, the highest TPC value is found in Moldavia region (413.3 *μ*g/mL), while in 2010 the TPC values are not very different. These differences could be explained by different weather conditions in these regions, because the TPC increases during the maturation of grapes, especially in warm and sunny areas [34]. The analysis of climatologic condition from 2008 and 2009 shows that during the maturation of grapes the temperatures were higher and sunny period was longer in Transylvania and Moldavia than in Oltenia and Muntenia, while the 2010 was a rainy year in all regions.

Statistical data analysis proves that the vineyard has a significant influence on the TPC (*p* = 0.003), while the varieties and vintage do not affect considerably the quantity of polyphenols, but a significant interaction effect between year and vineyard (*p* = 0.014) is observed. These results allow concluding that the polyphenolic content depends primarily on vineyard and winemaking technology, which is in agreement with other reported results [35].

### 3.2. Total Flavonoids Content (TFC)

The experimental values of TFC varied between 12.6 *μ*g/mL and 48.2 *μ*g/mL, being significantly influenced by variety (*p* = 0.032) and vineyard (*p* = 0.002), while the year does not have an important contribution to the variation of TFC (*p* = 0.977).

In the case of Sauvignon Blanc wines, the TFC values are different for Oltenia and Muntenia regions, the highest differences being between the two vineyards from Oltenia. The TFC is lower in vineyard I as compared to vineyard II, following the same trend during all three years.

The highest TFC value for Fetească Albă wines was obtained for the sample from Moldavia region in 2010 (41.68 *μ*g/mL). Differences between the two vineyards from Muntenia and, also, between Muntenia and Moldavia region were observed. The lowest TFC values correspond to wines from Muntenia vineyard II, followed by those from vineyard I whereas the highest TFC value belongs to Moldavia wines.

A descending trend of TFC values was observed over the years 2008–2010 for the Riesling wines from Transylvania and Oltenia regions, these values being higher than those for wines from Muntenia. Moreover, in the case of wines from Muntenia the situation is opposite; the TFC values increase from 2008 to 2010. The TFC value of Romanian Riesling wines is lower than the levels mentioned in the literature for this type of wine from other countries [36–38]; they even have the highest TFC content among the three studied wine types.

The average percent of flavonoids amount in total phenols was 9.38% being below the set level by 20% of TPC and proving the quality of the analyzed wines. The highest percentage is obtained for Riesling from Oltenia, 2008 (16.75%), and for Fetească Albă from Moldavia, 2010 (16.52%), while Sauvignon Blanc from Oltenia, 2010, has the smallest quantity of flavonoids in total phenols (5.06%).

### 3.3. Antioxidant Activity (AOA)

The experimental AOA values (Table 1) show that significant differences appear only from one vineyard to another (*p* = 0.000) and between varieties (*p* = 0.001). The year does not show significant effect on the AOA (*p* = 0.683), but a statistical interaction effect between year and vineyard (*p* = 0.002), respectively, and between year and variety (*p* = 0.035) is observed. For example, in the wines produced by the two vineyards from Oltenia region the AOA are ranging from 2.39 *μ*mol/mL to 3.43 *μ*mol/mL. The differences between vineyards could be due to the additional treatments used in the controlled fermentation of grapes that reduce the antioxidant activity as a result of chemical transformations, such as catechin derivatives oxidation. Also, the medium values of AOA show that Sauvignon Blanc wines have the smallest antioxidant activity (3.41 *μ*mol/mL), the highest activity being found for Riesling wines (4.65 *μ*mol/mL).

Analyzing the TPC and AOA values, it can be observed that a correlation between these two properties does not always exist. Thus, the wines from Muntenia region, produced in 2008 and 2009, and the wines produced in 2010 in the vineyard from Moldavia have a higher antioxidant activity comparing with polyphenolics content. In the case of wines from Transylvania a linear correlation between TPC and AOA is observed (*r* = 0.9992) proving that the antioxidant activity is determined only by polyphenolic compounds. The antioxidant activities are not correlated with polyphenolic content in the case of vineyards from Oltenia region. These behaviors are explained by the fact that the antioxidant activity depends more on the type of phenolic compounds from wine compared to their total quantity due to the following considerations: the relation structure-activity of antioxidant compounds; the contribution of each polyphenolic compound to the total antioxidant activity according to the number of OH and OCH_3_ groups and their position on the ring; the polymerization degree of phenolics; and the ratio between monomeric and polymeric forms because the inhibition of free radicals tends to increase with polymerization degree [18, 39].

### 3.4. Total Sugars Content (TSC)

The TSC is an important regulatory parameter that is used to classify wine styles and to determine the endpoint of fermentation, the sugars being responsible for the formation of ethanol as well as a number of secondary products [40].

Experimental results show that Fetească Albă wines from the region of Moldavia are semisweet wines. In 2008, the potential of sugars accumulation is higher than that in 2009 and 2010, the TSC being highest in this year (49.01 mg/mL). This fact is due to climatologic conditions, on that year being low precipitation and high temperature during ripening, which have led to a high accumulation of sugars. This type of wines originating from the Muntenia region is found to have a high content of sugars; the quantity is different among vineyards, being higher in vineyard I than in vineyard II. This trend is valid for all three years. This behavior could be explained by differences in the winemaking technology, particularly fermentation process that was used in the vineyards.

Regarding the Sauvignon Blanc wines, the trend of TSC is different among Oltenia and Muntenia region. In Oltenia region, the highest value is obtained in the 2008 year in both vineyards, while the TSC values for 2009 and 2010 are almost equal. On the other hand, in Muntenia region the highest TSC value is obtained in 2010, whereas for 2008 and 2009 the obtained TSC values are very similar.

In the case of Riesling wines, the highest TSC corresponds to the year 2008 for all three regions, being much higher compared to 2009 and 2010. In the Oltenia region, the year 2008 proved to be the most favorable one regarding climatic conditions for the accumulation of sugars (7.86 g/L). In the following years, the trend is descending, the TSC values significantly decreasing until 5.63 g/L in 2009, respectively, and 4.52 g/L in 2010. On the other hand, in Transylvania and Muntenia, the situation is opposite; the lowest TSC values correspond to 2009 and slow increase of TSC is observed in 2010.

From ANOVA analysis it can be seen that the variety and the vineyard have a significant influence on the TSC (*p* = 0.024, *p* = 0.000), while the vintage does not affect considerably the quantity of sugars, but a significant interaction effect between year and vineyard (*p* = 0.005) is observed.

One of the factors that could be responsible for the TSC modification of TSC from one region to another is the water deficit, which is one of the main components of the so-called “terroir effect.” The water deficit leads to a low dimension of berry, thus influencing the sugar content due to the limited carbon assimilation. However, a slight-to-moderate water deficit has a positive effect on the berries and wine quality because the content of sugars is higher in smaller berries than in bigger ones, where a dilution of sugars has been produced [41].

### 3.5. pH

The determined pH values are between 3.03 and 3.46 being strongly influenced by variety of wine (*p* = 0.002), whereas the vineyard has a weak influence on it (*p* = 0.049). The pH values of Sauvignon Blanc wines are different in the two regions, Oltenia and Muntenia. Also, the pH of the wines from Oltenia is higher for those from vineyard I compared to those from vineyard II. Regarding Fetească Albă wines, no large variations in pH were observed for 2009 and 2010, but some differences were observed between vineyards and regions. Moreover, even in the same region, namely, Muntenia, the pH values varied between vineyards being lower in vineyard I than in vineyard II where the wines have the highest pH values. In the case of Riesling sort, a descending trend of pH was observed over the years 2008–2010 for the wines from Transylvania region. Over against Transylvania, the pH values of wines from Muntenia showed an ascending tendency over these three years.

The pH has an effect on stability of wine, low pH inhibiting microorganism growth. As a result, white wine with a pH above 3.4 and red wine above 3.5 may have stability problems [42]. The experimental results show that two wines, namely, Fetească Albă from vineyard II, Muntenia, 2009 and 2010 vintage, exceed slightly the value of 3.4 and, consequently, could have some problems regarding their stability.

### 3.6. Isotopic Analysis

From the isotopic values of investigated wines presented in Table 1, a statistical interaction effect between year and vineyard was observed (*p* = 0.004). The isotopic differences which appear in the same wine sort, produced in the same area but in different production years, are due to the different meteorological condition that prevails in the above-mentioned years. For instance, in the case of Sauvignon Blanc wine from Oltenia, produced in the same vineyard in 2008, 2009, and 2010, the oxygen isotopic value is varying from *δ* ^18^O = 3.7‰ (for 2008 vintage) to *δ* ^18^O = 1.1‰ (for 2009 vintage) and *δ* ^18^O = 1.6‰ (for 2010 vintage), respectively. These differences arise mainly from the different quantities of precipitation that had been fallen in these years, rather than important differences in temperature among these years. Thus, the annual mean temperature of all three years in this area was about 12°C with slightly higher temperature in 2008 during the maturation period; meanwhile, the quantity of precipitation in 2008 was 592 mm/year comparative with 2009 and 2010 vintages when the quantity of precipitations was 674 mm/year and 708 mm/year, respectively.

Even if 2009 was a warm and dry year in Transylvania region, *δ* ^18^O value of the wine that was produced in this year is the lowest one, *δ* ^18^O = −1.2‰, as compared with those from 2008 and 2010, respectively (Table 1). In this case, it is necessary to take into account the fact that during the harvesting period there were significant precipitations falls, which can affect drastically the isotopic ratio of oxygen. In this sense, important variation of oxygen isotopic ratios as function of harvesting date was previously reported by [43].

From Muntenia region, the investigated wine sorts were as follows: Sauvignon Blanc, Fetească Albă, and Riesling. In this region, the lowest *δ* ^18^O values were obtained for the wines produced in the rainy year 2010. Beside this, the warm and dry year 2009 was characterized by higher values of *δ* ^18^O from wine water especially in comparison with those from 2008 harvest.

The investigation of two wine sorts from the same vineyard from Oltenia region presents the same trend of *δ* ^18^O variation from one year to another; moreover *δ* ^18^O values of the wine produced in the same year are very similar. This can be explained by the fact that *δ* ^18^O is a better indicator for geographical origin rather than for identification of wine sort.

The most important factor that affects wine water *δ* ^18^O value is the isotopic composition of the water which is available to plant. In most cases, this available plant water is the rain water, which is directly related to the meteorological particularities of a specific year. It was previously reported that the degree of isotopic enrichment among different fruits (or sorts of the same fruits) is species dependent [44]. On the other hand, different harvest periods, specific for each grape sort, will generate specific *δ* ^18^O values for each wine type. Thus, we can explain the interdependence between the production year and the wine sort.

### 3.7. Statistical Analysis

The differentiation and classification of wine samples on the basis of their chemical composition, geographical origin, variety, or quality belong to the basic applications of chemometric methods in enology, methods that offer the possibility of a fast and efficient extraction of the information originating from large sets of data [40].

The ANOVA is the initial step in the identification of some parameters that are statistically contributing to the data sets variability providing information on the differences among wine varieties or wines from different regions. The importance of the parameter is greater if *p* value is lower. Also, the importance of each characteristic of wine can be ranked on the basis of *p* values. The most important parameter that can distinguish between the wine varieties is the AOA followed closely by pH. TSC and TFC have a moderate influence, while TPC cannot be used for differentiation of wines. Regarding the vineyard the best variables to discriminate between wine samples were AOA and TSC followed closely by TFC and TPC, whereas the pH has a moderate significance in the wine discrimination. However, unlike varieties and region, the vintage cannot distinguish the wine samples, all *p* values being greater than 0.05. It is also clear that *δ* ^18^O values are not among the significant characteristics for wines differentiation according to variety, vineyard, or vintage. In conclusion, ANOVA enabled the discrimination among origins of wines, without being able to conclude which parameters are the best descriptors. The resulting elements will always depend on the combination of varieties under investigation [26].

**Figure 1:**
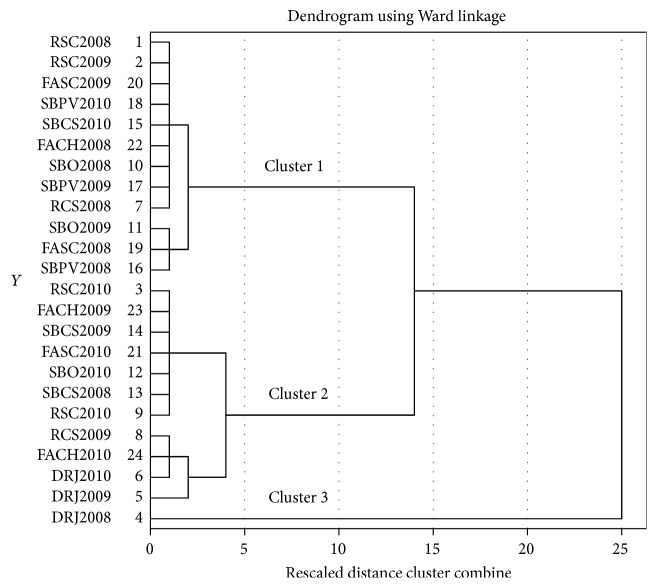
The obtained dendrogram of CLU analysis using Ward's method.

Cluster analysis is an unsupervised pattern recognition that involves trying to determine relationships between objects (samples) without using any prior information about these relationships [45]. Hierarchical agglomerative cluster analysis is the most common approach and its result is typically illustrated by a dendrogram. Generally, the objects have similar properties within a cluster and different proprieties between clusters. As a clustering criterion Ward's method was used, with Square Euclidian distance as a measure interval between groups. The obtained dendrogram is presented in Figure 1. The twenty-seven wine samples were divided into three main clusters. In order to find out the differences between the three clusters, ANOVA was run again, this time having as independent variable the grouping cluster variable, obtained from previous analysis. From the six parameters used in statistical analysis, only TPC, TFC, and *δ* ^18^O have made a distinction between the three clusters. Cluster 1 comprises wine samples from Muntenia and Oltenia region; meanwhile, in cluster 2 an overlay appears from all three wine regions. Finally, cluster 3 comprises only one sample from Transylvania region, DRJ 2008, with the highest TPC and TFC values.

PCA was applied to entire data set containing all measured parameters. The obtained value of KMO test is 0.581, which indicates that PCA is suitable for carrying out the analysis of our data set. The value obtained for Bartlett test (0.001) also indicates that some relationship between the variables exists and the PCA is appropriate for data analysis. The correlation matrix (Table 2) was used for analysis, where the values greater than 0.3 representing a strong correlation between variables are marked with italics. Significantly positive correlations were found between TFC and AOA, TFC and TPC, AOA and TPC, respectively, and TSC and TFC. Some negative correlations were also observed between *δ* ^18^O and TPC, *δ* ^18^O and TFC, respectively, and pH and TFC. These strong correlations, which were observed between analyzed variables, are another indicator that PCA is appropriate and might provide reliable and distinct principal components.

PCA was running using the following parameters: extraction method, principal component, and eigenvalues, and values greater than 1 were retained. The results indicate that only two components have eigenvalues bigger than 1 (2.462 and 1.400, resp.). The first component explains 41.04% from the total variance, while the second component has a variance of 23.33%. The first two components retained explain a total variance of 64.37%.

**Figure 2:**
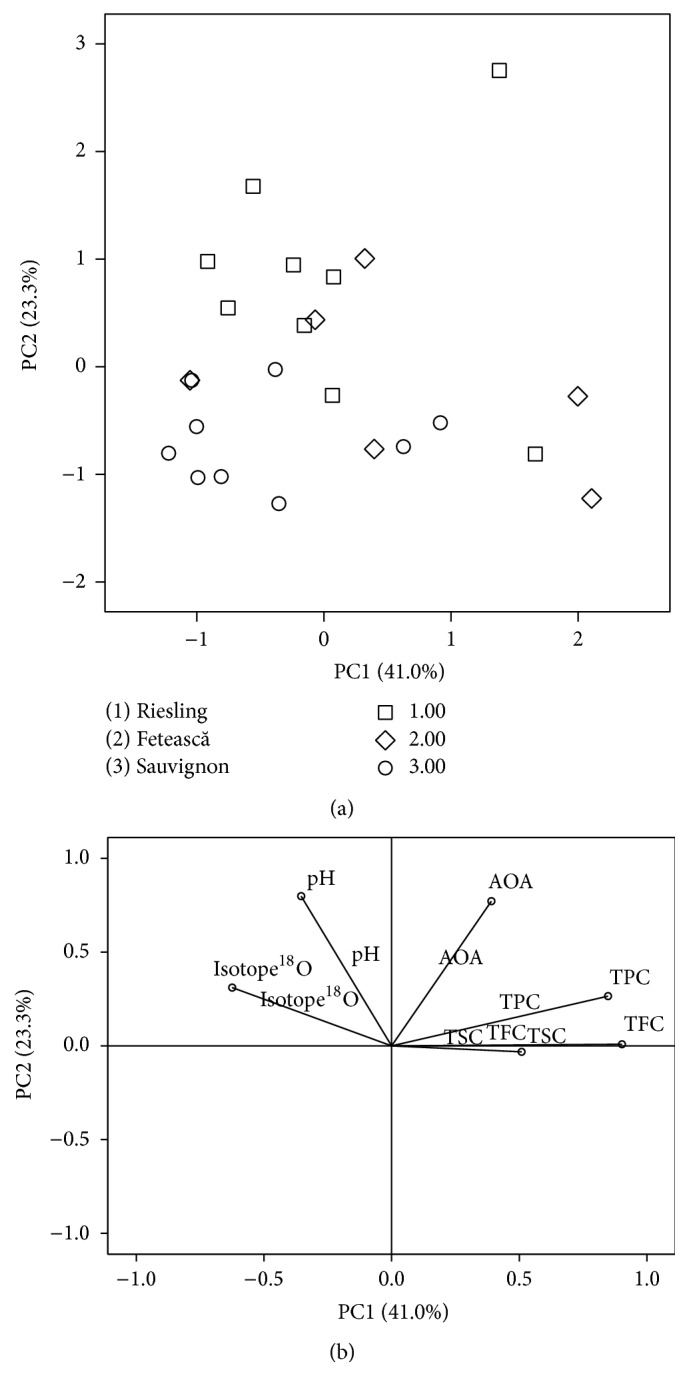
Score of PCA (a) and the PCA loadings (b) for the tested wine samples.

The loadings of each principal component extracted are presented in Table 3. The first principal component has strong positive loading of TPC and TFC (values > 0.75), moderate loading on TSC (values between 0.5 and 0.75), and weak loading on AOA (values of 0.3–0.5), while the second one has strong positive loading of AOA and pH and weak loading of isotope ratios ^18^O/^16^O. As a result, the most important PCA graph is obtained (Figure 2(a)), where the natural grouping of the analyzed wine samples can be observed. Most of the Sauvignon variety samples are well separated from Riesling and Fetească and had negative PC1 and PC2 values. The majority of Riesling samples have negative values for PC1 and positive values for PC2. Some of Fetească samples had different PC values, compared to other samples of the same variety. The variability among the same-sort wine samples is due to the fact that wines come from different regions of origin, thus not only variety but also region affects the positioning of the wine samples. These results are analogous with those obtained by other authors [26].

Figure 2(b) shows the loadings plot in PC1-PC2 plan. Large PC1 positive values were observed for AOA, TPC, and TFC. In contrast, the pH and isotope ratio ^18^O/^16^O exhibit negative values for PC1. With regard to the PC2, only TSC display slightly negative value, while all other parameters have large positive values.

## 4. Conclusion

The analyzed white wines present differences with regard to cultivars, vineyard, and vintage. The results show that the tested parameters together with chemometric techniques have a significant potential to be used to discriminate white wines. Some of the tested parameters of wines (TPC, TFC, AOA, and TSC) are significantly influenced by the vineyard, whereas cultivars exerted significant influence on TFC, AOA, TSC, and pH. The vintage has no influence on the wines characteristics, but it presents a significant interaction with vineyard in the case of TPC, AOA, TSC, and *δ* ^18^O and with cultivars in the case of AOA. Also, it has been demonstrated that chemometric methods used in wine samples interpretation are very useful in order to highlight the essential parameters and to classify the sample after a predetermined criterion.

## Figures and Tables

**Table 1:** The experimental values of determined parameters of investigated white wines.

Number	Wine cultivars	Viticultural area	Production year	TPC (*μ*g/mL)	TFC (*μ*g/mL)	AOA (*μ*mol/mL)	TSC (mg/mL)	pH	*δ* ^18^O
1	Sauvignon Blanc	Oltenia, vineyard I	2008	210.7 ± 17.6	15.1 ± 0.5	2.84 ± 0.05	5.08 ± 0.10	3.23	3.7
2			2009	174.8 ± 5.7	16.4 ± 1.3	2.65 ± 0.04	4.82 ± 0.10	3.23	1.1
3			2010	248.5 ± 18.3	12.6 ± 0.9	2.39 ± 0.05	4.81 ± 0.07	3.16	1.6
4		Oltenia, vineyard II	2008	237.8 ± 18.5	21.5 ± 1.1	3.44 ± 0.02	5.17 ± 0.08	3.06	1.7
5			2009	257.4 ± 7.4	28.2 ± 5.9	3.78 ± 0.02	3.87 ± 0.05	3.12	2.1
6			2010	201.9 ± 11.9	19.9 ± 0.5	3.43 ± 0.04	4.04 ± 0.09	3.11	1.1
7		Muntenia, vineyard I	2008	189.6 ± 9.0	16.2 ± 0.2	3.34 ± 0.11	4.65 ± 0.11	3.11	2.8
8			2009	211.9 ± 6.5	21.4 ± 1.9	4.36 ± 0.04	4.72 ± 0.10	3.24	3.4
9			2010	221.5 ± 8.6	19.4 ± 5.0	4.49 ± 0.11	5.61 ± 0.13	3.32	2.4

10	Fetească Albă	Muntenia, vineyard I	2008	204.4 ± 8.4	19.6 ± 0.5	3.32 ± 0.03	8.00 ± 0.15	3.08	2.5
11			2009	260 ± 4.0	25.2 ± 9.0	3.56 ± 0.03	7.90 ± 0.13	3.25	1.5
12			2010	294.8 ± 8.4	23.9 ± 0.7	4.48 ± 0.03	7.14 ± 0.13	3.26	−0.7
13		Muntenia, vineyard II	2008	176.7 ± 18.6	13.6 ± 0.5	4.72 ± 0.05	6.69 ± 0.13	3.19	3.0
14			2009	230.7 ± 3.6	13.6 ± 0.8	4.85 ± 0.04	4.93 ± 0.08	3.46	3.9
15			2010	261.5 ± 21.4	16.7 ± 2.2	5.49 ± 0.06	5.00 ± 0.06	3.42	1.4
16		Moldavia	2008	326.7 ± 15.4	25.4 ± 5.1	3.95 ± 0.03	49.01 ± 0.35	3.23	4.2
17			2009	413.3 ± 9.6	24.9 ± 8.8	4.96 ± 0.13	32.32 ± 0.45	3.32	−3.3
18			2010	252.2 ± 9.1	41.7 ± 0.9	5.42 ± 0.04	35.68 ± 0.40	3.32	6.2

19	Riesling	Muntenia, vineyard II	2008	228.1 ± 8.6	18.5 ± 1.8	4.92 ± 0.02	5.78 ± 0.11	3.04	3.5
20			2009	227.8 ± 13.7	20.1 ± 3.1	5.14 ± 0.03	4.67 ± 0.09	3.32	4.1
21			2010	261.9 ± 4.2	24.1 ± 3.5	5.33 ± 0.04	5.10 ± 0.08	3.11	−0.8
22		Transylvania	2008	445.2 ± 6.1	48.2 ± 8.0	5.20 ± 0.01	7.38 ± 0.09	3.21	1.7
23			2009	339.6 ± 22.8	32.1 ± 3.1	4.43 ± 0.08	5.66 ± 0.07	3.06	−1.2
24			2010	276.3 ± 0.6	26.4 ± 0.9	3.90 ± 0.02	6.00 ± 0.07	3.03	1.8
25		Oltenia, vineyard II	2008	208.1 ± 18.9	34.9 ± 10.9	4.21 ± 0.05	7.83 ± 0.07	3.03	1.3
26			2009	293 ± 11.6	32.4 ± 0.1	4.54 ± 0.04	5.63 ± 0.06	3.04	1.8
27			2010	247.8 ± 7.8	31.7 ± 1.8	4.19 ± 0.04	4.52 ± 0.09	3.16	1.1

**Table 2:** The correlation matrix.

	AOA	TPC	TFC	*δ* ^18^O	TSC	pH
AOA	1.000					
TPC	*0.397*	1.000				
TFC	*0.318*	*0.760*	1.000			
*δ* ^18^O	−0.034	*−0.416*	*−0.391*	1.000		
TSC	0.120	0.264	*0.363*	−0.152	1.000	
pH	0.291	−0.042	*−0.342*	0.293	−0.138	1.000

The italic values correspond to statistically significant correlation.

**Table 3:** Loading of two-factor model that explains 64.37% of the total variance.

Variable	PC1	PC2
AOA	0.421	0.756
TPC	0.858	0.232
TFC	0.902	−0.027
Isotope ratios ^18^O/^16^O	−0.612	0.335
TSC	0.508	−0.052
pH	−0.323	0.812
